# Genetic risk score of common genetic variants for impaired fasting glucose and newly diagnosed type 2 diabetes influences oxidative stress

**DOI:** 10.1038/s41598-018-26106-z

**Published:** 2018-05-18

**Authors:** Minjoo Kim, Minkyung Kim, Limin Huang, Sun Ha Jee, Jong Ho Lee

**Affiliations:** 1 Research Center for Silver Science, Institute of Symbiotic Life-TECH, Yonsei University, Seoul, 03722 Korea; 2 Department of Food and Nutrition, Brain Korea 21 PLUS Project, College of Human Ecology, Yonsei University, Seoul, 03722 Korea; 3 National Leading Research Laboratory of Clinical Nutrigenetics/Nutrigenomics, Department of Food and Nutrition, College of Human Ecology, Yonsei University, Seoul, 037222 Korea; 4 Institute for Health Promotion, Graduate School of Public Health, Yonsei University, Seoul, 03722 Korea

## Abstract

We tested the hypothesis that the cumulative effects of common genetic variants related to elevated fasting glucose are collectively associated with oxidative stress. Using 25 single nucleotide polymorphisms (SNPs), a weighted genetic risk score (wGRS) was constructed by summing nine risk alleles based on nominal significance and a consistent effect direction in 1,395 controls and 718 patients with impaired fasting glucose (IFG) or newly diagnosed type 2 diabetes. All the participants were divided into the following three groups: low-wGRS, middle-wGRS, and high-wGRS groups. Among the nine SNPs, five SNPs were significantly associated with IFG and type 2 diabetes in this Korean population. wGRS was significantly associated with increased IFG and newly diagnosed type 2 diabetes (*p* = 6.83 × 10^−14^, odds ratio = 1.839) after adjusting for confounding factors. Among the IFG and type 2 diabetes patients, the fasting serum glucose and HbA_1c_ levels were significantly higher in the high-wGRS group than in the other groups. The urinary 8-epi-PGF_2α_ and malondialdehyde concentrations were significantly higher in the high-wGRS group than in the other groups. Moreover, general population-level instrumental variable estimation (using wGRS as an instrument) strengthened the causal effect regarding the largely adverse influence of high levels of fasting serum glucose on markers of oxidative stress in the Korean population. Thus, the combination of common genetic variants with small effects on IFG and newly diagnosed type 2 diabetes are significantly associated with oxidative stress.

## Introduction

Oxidative stress has been reported as a contributing factor for the development of type 2 diabetes, diabetic complications and cardiovascular diseases^1^. Increased oxidative stress is associated with insulin resistance (IR), dyslipidemia, β-cell dysfunction, impaired fasting glucose (IFG), impaired glucose tolerance (IGT) and, ultimately, type 2 diabetes. Isoprostanes, which are derived from polyunsaturated fatty acids, are the most valuable biomarkers of oxidative stress and lipid peroxidation in biological systems^2^. Isoprostane assays measure the urinary levels of F_2_-isoprostanes, which become elevated in response to arachidonic acid peroxidation. Among these products, 8-epi-prostaglandin F_2α_ (8-epi-PGF_2α_) is particularly important because it is the best-studied F_2_-isoprostane. According to previous studies, 8-epi-PGF_2α_ is a predictor of glycemic control and oxidation status in patients with type 2 diabetes^3^. In addition, 8-epi-PGF_2α_ is a reliable marker of IGT^4^.

The genetic risk score (GRS) represents an integrative analytical approach for type 2 diabetes risk prediction that can be used efficiently and effectively to construct genome-wide risk measures based on the findings of genome-wide association studies (GWAS)^5–7^. Recent genetic risk assessment studies of type 2 diabetes have evaluated the predictive value of cumulative genetic scores^8,9^. The further development and improvement of a multi-single nucleotide polymorphism (SNP) GRS have led to better disease prediction and prevention in an independent ethnic population. We assessed the likelihood that currently available genetic information could be used to enhance disease prediction by constructing a GRS selected from 35 susceptibility variants for elevated fasting glucose^10^ to test the hypothesis that the cumulative effects of these common genetic variants of IFG and type 2 diabetes are associated with oxidative stress. To strengthen this study, we also performed a Mendelian randomization analysis to investigate the causal effects of fasting serum glucose on markers of oxidative stress.

## Results

The IFG and newly diagnosed type 2 diabetes patients (*n* = 718) included a significantly higher proportion of males who were older and heavier than the normal fasting glucose (NFG) controls (*n* = 1,395). The IFG and type 2 diabetes patients also exhibited significantly higher systolic and diastolic blood pressure (BP), triglyceride levels, fasting serum glucose levels, homeostatic model assessment of IR (HOMA-IR) scores, and hemoglobin A_1c_ (HbA_1c_) levels than the controls. In addition, the subjects with IFG and newly diagnosed type 2 diabetes presented higher levels of urinary 8-epi-PGF_2α_ and plasma malondialdehyde (MDA) than the controls (Table 1). The allelic frequencies of the 25 SNPs are shown in Table 2.

**Table 1:** Clinical characteristics of the study participants.

	NFG (*n* = 1,395)	IFG and type 2 diabetes (*n* = 718)	*p*-value	*p′*-value
Sex (male/female)	482/913	374/344	<0.001	—
Age (year)	48.0 ± 0.31	52.8 ± 0.40	<0.001	—
BMI (kg/g)	23.7 ± 0.08	25.0 ± 0.11	<0.001	—
Waist-to-hip ratio	0.88 ± 0.00	0.90 ± 0.00	<0.001	0.368
Systolic BP (mmHg)	119.6 ± 0.41	126.8 ± 0.60	<0.001	<0.001
Diastolic BP (mmHg)	75.2 ± 0.30	79.0 ± 0.40	<0.001	0.013
Triglyceride (mg/dL)^*§*^	118.1 ± 1.90	144.3 ± 3.34	<0.001	0.001
Total cholesterol (mg/dL)^§^	197.3 ± 0.95	199.3 ± 1.38	0.314	0.458
HDL cholesterol (mg/dL)^§^	54.2 ± 0.35	50.6 ± 0.50	<0.001	0.068
LDL cholesterol (mg/dL)^§^	119.9 ± 0.86	121.6 ± 1.28	0.418	0.304
Fasting serum glucose (mg/dL)^§^	86.9 ± 0.20	118.4 ± 0.97	<0.001	<0.001
Insulin (μIU/dL)^§^	9.09 ± 0.11	9.68 ± 0.24	0.301	0.674
HOMA-IR^§^	1.96 ± 0.03	2.85 ± 0.08	<0.001	<0.001
HbA_1c_ (%)^§^	5.68 ± 0.02	6.52 ± 0.05	<0.001	<0.001
8-epi-PGF_2α_ (pg/mg creatinine)^§^	1497.3 ± 24.0	1761.4 ± 35.5	<0.001	<0.001
Malondialdehyde (nmol/mL)^§^	8.21 ± 0.06	11.8 ± 0.24	<0.001	<0.001

Mean ± SE. ^*§*^Tested via logarithmic transformation. The *p*-value was derived from an independent *t*-test between two groups. The *p′*-value was derived from an independent *t*-test between two groups after adjusting for age, sex, and BMI.

**Table 2:** Association of 25 SNP loci with IFG and type 2 diabetes in a Korean population.

SNP	Nearby gene^a^	Risk allele^b^	RAF (case/control)	Unadjusted		Adjusted^c^	
				*p*-value	OR (95% CI)	*p*-value	OR (95% CI)
rs340874	*PROX1*	C	0.654/0.370	0.991	1.001 (0.878–1.140)	0.957	1.004 (0.874–1.153)
rs1260326	*GCKR*	C	0.487/0.420	**0**.**000028**	1.331 (1.155–1.488)	**0**.**000009**	1.356 (1.185–1.550)
rs11708067	*ADCY5*	A	0.999/0.997	0.184	4.090 (0.511–32.730)	0.143	4.970 (0.582–42.415)
rs7756992	*CDKAL1*	G	0.580/0.561	0.228	1.082 (0.952–1.229)	0.082	1.128 (0.985–1.291)
rs9368222	*CDKAL1*	A	0.510/0.485	0.130	1.103 (0.972–1.251)	0.060	1.137 (0.995–1.300)
rs7747752	*CDKAL1*	C	0.560/0.541	0.234	1.080 (0.951–1.226)	0.089	1.124 (0.982–1.285)
rs2191349	*DGKB*	T	0.700/0.666	**0**.**023**	1.171 (1.022–1.342)	**0**.**011**	1.207 (1.045–1.394)
rs1799884	*GCK*	T	0.208/0.175	**0**.**009**	1.237 (1.055–1.450)	**0**.**027**	1.209 (1.022–1.430)
rs3757840	*GCK*	T	0.619/0.596	0.149	1.101 (0.966–1.254)	0.356	1.067 (0.930–1.224)
rs4607517	*GCK*	A	0.246/0.213	**0**.**014**	1.204 (1.038–1.398)	**0**.**045**	1.175 (1.004–1.375)
rs11558471	*SLC30A8*	A	0.627/0.581	**0**.**004**	1.211 (1.064–1.378)	**0**.**002**	1.240 (1.082–1.422)
rs7034200	*GLIS3*	A	0.427/0.408	0.233	1.081 (0.951–1.228)	0.216	1.089 (0.951–1.247)
rs10811661	*CDKN2A/B*	T	0.606/0.537	**0**.**000017**	1.325 (1.166–1.507)	**0**.**000010**	1.358 (1.186–1.555)
rs4506565	*TCF7L2*	T	0.031/0.026	0.294	1.221 (0.841–1.774)	0.241	1.268 (0.853–1.886)
rs7903146	*TCF7L2*	T	0.035/0.028	0.197	1.265 (0.885–1.808)	0.119	1.353 (0.925–1.977)
rs12243326	*TCF7L2*	C	0.003/0.002	0.860	1.117 (0.327–3.823)	0.968	0.972 (0.240–3.938)
rs11603334	*ARAP1*	G	0.947/0.940	0.324	1.149 (0.872–1.515)	0.601	1.081 (0.807–1.447)
rs1387153	*MTNR1B*	T	0.466/0.414	**0**.**001**	1.236 (1.088–1.403)	**0**.**000109**	1.304 (1.140–1.492)
rs2166706	*MTNR1B*	C	0.475/0.418	**0**.**00039**	1.258 (1.108–1.429)	**0**.**000041**	1.326 (1.159–1.517)
rs10830963	*MTNR1B*	G	0.482/0.432	**0**.**002**	1.224 (1.078–1.389)	**0**.**000089**	1.309 (1.144–1.497)
rs2293941	*PDX1*	A	0.458/0.476	0.269	0.931 (0.820–1.057)	0.209	0.918 (0.803–1.049)
rs17271305	*FAM148B/VPS13C/C2CD4A/B*	G	0.158/0.179	0.118	0.865 (0.720–1.068)	0.076	0.840 (0.693–1.018)
rs4502156	*FAM148B/VPS13C/C2CD4A/B*	T	0.460/0.437	0.163	1.094 (0.964–1.242)	0.108	1.116 (0.976–1.276)
rs11071657	*FAM148B/VPS13C/C2CD4A/B*	A	0.658/0.654	0.768	1.020 (0.893–1.166)	0.896	1.009 (0.877–1.162)
rs10423928	*GIPR*	A	0.214/0.198	0.220	1.102 (0.994–1.288)	0.208	1.111 (0.943–1.309)
wGRS				**2**.**4364E-12**	1.712 (1.473–1.990)	**6**.**8265E-14**	1.839 (1.568–2.157)

The results of logistic regression analysis are shown.

OR, odds ratio; 95% CI, 95% confidence interval; wGRS, weighted genetic risk score.

^a^Information in the original report is shown.

^b^Risk allele reported in previous reports.

^c^Adjusted for age, sex, and BMI.

wGRS was calculated including SNPs with nominal significance (*p* < 0.05).

### Association of each SNP with IFG and newly diagnosed type 2 diabetes

Using 25 SNPs from 15 loci, a weighted genetic risk score (wGRS) was constructed using nine association signals based on nominal significance and a consistent effect direction in a Korean population (Table 2). Five SNPs (*GCKR* rs1260326, *CDKN2A/B* rs10811661, *MTNR1B* rs1387153, rs2166706, and rs10830963) were significantly associated with IFG and type 2 diabetes (*p* = 9.0 × 10^−6^, 1.0 × 10^−5^, 1.09 × 10^−4^, 4.1 × 10^−5^, and 8.9 × 10^−5^, respectively) after adjusting for age, sex, and body mass index (BMI), and rs1260326 in *GCKR* exhibited the strongest association in the Korean sample. Additionally, rs2191349 in *DGKB*, rs1799884 and rs4607517 in *GCK*, and rs11558471 in *SLC30A8* were nominally associated with IFG and type 2 diabetes (*p* = 0.011, 0.027, 0.045, and 0.002, respectively) after adjusting for age, sex, and BMI. Moreover, wGRS was significantly associated with increased IFG and newly diagnosed type 2 diabetes [*p* = 6.83 × 10^−14^, odds ratio (OR) per risk allele = 1.839, 95% confidence interval (CI) = 1.568–2.157] after adjusting for age, sex, and BMI (Table 2). According to the weighted tertile analysis, an increased number of risk alleles were associated with an increased OR for IFG and type 2 diabetes risk (Supplementary Figure S1).

### Associations between wGRS and clinical characteristics

The IFG and type 2 diabetes patients exhibited a significantly higher wGRS than the NFG controls (1.93 ± 0.02 vs. 1.73 ± 0.02, *p* < 0.001) both before and after adjusting for age, sex, and BMI. Among the total study participants, wGRS was positively correlated with fasting serum glucose (*r* = 0.177, *p* < 0.001), HbA_1c_ (*r* = 0.169, *p* < 0.001), HOMA-IR (*r* = 0.078, *p* < 0.001), 8-epi-PGF_2α_ (*r* = 0.101, *p* < 0.001), and MDA (*r* = 0.134, *p* < 0.001). In the NFG controls, wGRS was positively correlated with fasting serum glucose (*r* = 0.126, *p* < 0.001). In the IFG and type 2 diabetes patients, HbA_1c_ (*r* = 0.153, *p* = 0.004), HOMA-IR (*r* = 0.087, *p* = 0.023), 8-epi-PGF_2α_ (*r* = 0.197, *p* < 0.001), and MDA (*r* = 0.231, *p* < 0.001) showed significant positive correlations with wGRS, and fasting serum glucose tended to be correlated with wGRS (*r* = 0.072, *p* = 0.055).

### Investigation of the combined effects of wGRS on clinical features and oxidative stress

**Figure 1:**
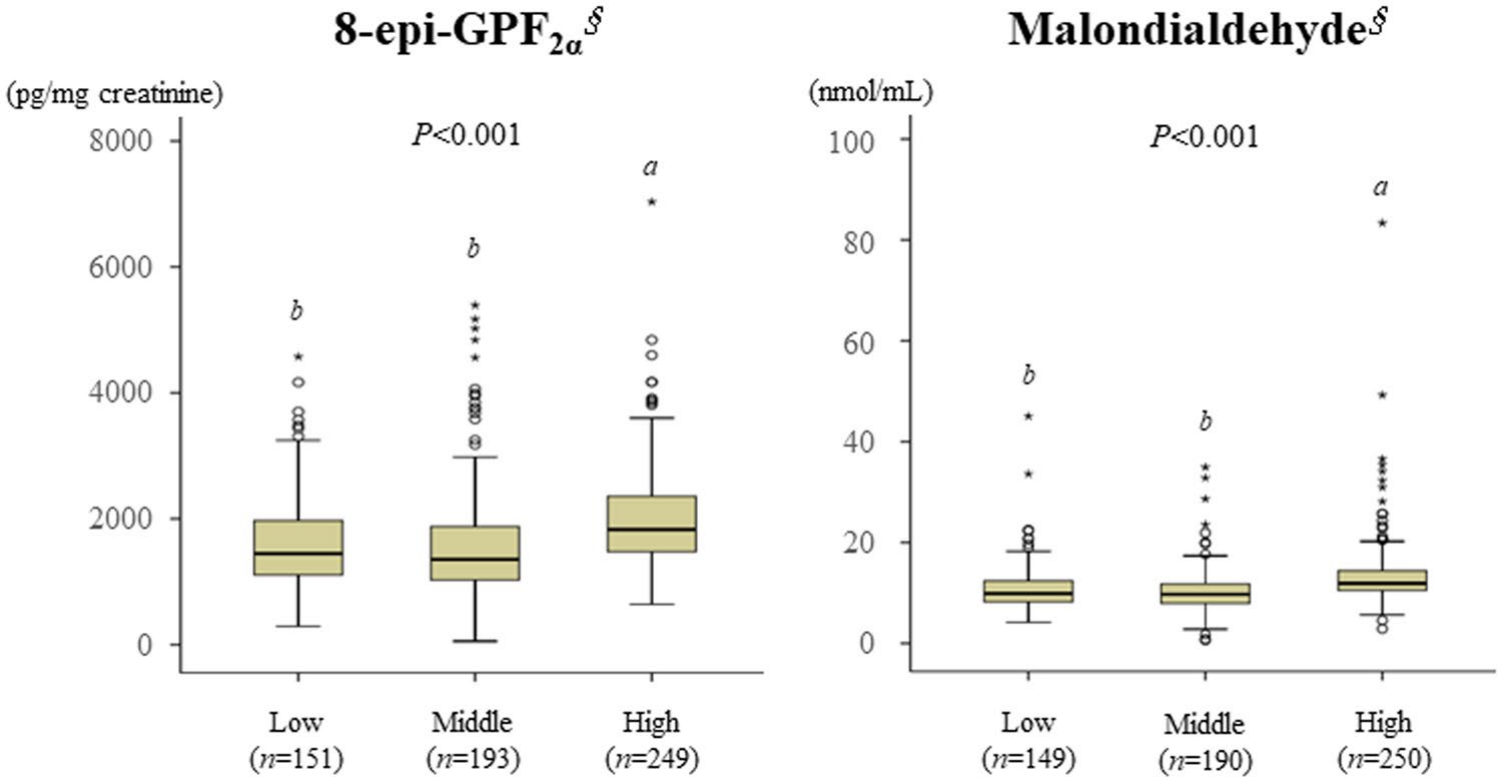
Differences in 8-epi-GPF_2α_ and malondialdehyde concentrations among the three groups of IFG and type 2 diabetes patients. Mean ± SE. ^∮^Tested via logarithmic transformation. One-way ANOVA was performed to calculate *p*-values. All letters indicating *p* < 0.05 were derived from Bonferroni’s *post hoc* test. Comparisons without a significant difference are indicated by the same letter, and significant differences are indicated by different letters.

All the participants were divided into the following three approximately equally sized strata according to the wGRS, as described in Supplementary Table S1: low-wGRS, middle-wGRS, and high-wGRS groups. The characteristics of the IFG and type 2 diabetes patients in the three groups are shown in Table 3. The fasting serum glucose and HbA_1c_ levels of the high-wGRS group were significantly higher than those of the low- and middle-wGRS groups. The HOMA-IR score was also significantly higher in the high-wGRS group than in the low-wGRS group. The urinary 8-epi-PGF_2α_ and plasma MDA concentrations recorded in the IFG and type 2 diabetes patients in the three groups are shown in Fig. 1. The urinary 8-epi-PGF_2α_ and plasma MDA levels were significantly higher in the high-wGRS group than in the low- and middle-wGRS groups. The characteristics of the NFG control subjects in the three groups are shown in Supplementary Table S2. The fasting serum glucose level in the high-wGRS group was significantly higher than that in the low-wGRS group. Among the total study participants, the fasting serum glucose, HbA_1c_, HOMA-IR, urinary 8-epi-PGF_2α_, and plasma MDA concentrations were significantly different between the different wGRS groups (Supplementary Table S3).

**Table 3:** Characteristics of the IFG and type 2 diabetes groups subdivided by the wGRS into three groups.

	Low wGRS (*n* = 183)	Middle wGRS (*n* = 227)	High wGRS (*n* = 308)	*p*-value
Age (year)	53.0 ± 0.88	52.8 ± 0.66	52.6 ± 0.59	0.928
BMI (kg/m^2^)	25.1 ± 0.22	24.9 ± 0.21	25.1 ± 0.17	0.744
Waist-to-hip ratio	0.91 ± 0.00	0.90 ± 0.00	0.90 ± 0.00	0.367
Fasting serum glucose (mg/dL)^§^	116.4 ± 2.10^b^	116.7 ± 1.74^b^	120.7 ± 1.37^*a*^	0.011
Insulin (μIU/dL)^§^	8.80 ± 0.31	9.57 ± 0.36	10.3 ± 0.45	0.082
HbA_1c_ (%)^§^	6.37 ± 0.08^b^	6.38 ± 0.09^*b*^	6.73 ± 0.08^*a*^	<0.001
HOMA-IR^§^	2.52 ± 0.10^b^	2.73 ± 0.11^*a*,*b*^	3.13 ± 0.17^*a*^	0.008

Mean ± SE. ^*§*^Tested via logarithmic transformation. The *p*-value was derived from a one-way ANOVA among the three groups. All letters indicating *p* < 0.05 were derived from Bonferroni’s *post hoc* test. Comparisons without a significant difference are indicated by the same letter, and significant differences are indicated by different letters.

### Causal estimates from a Mendelian randomization analysis

The causal effects of fasting serum glucose on markers of oxidative stress were inferred through instrumental variable (IV) estimation techniques. The corresponding causal relationships were also assessed based on the association of wGRS with markers of oxidative stress (Supplementary Table S4). General population-level causal effects were assessed as IV estimates, where wGRS was used as an IV in the total study participants (Supplementary Table S5). As a result, one unit of fasting serum glucose (mg/dL) was associated with higher levels of oxidative stress markers, including urinary 8-epi-PGF_2α_ and plasma MDA (all *p* < 0.001).

### Association between specific SNPs and markers of oxidative stress

Based on the results shown in Table 2, we performed a multiple regression analysis to determine the direct contributors to urinary 8-epi-PGF_2α_ and plasma MDA. rs2191349 (standardized *β* = 0.067, *p* = 0.004) and rs10811661 (standardized *β* = 0.061, *p* = 0.008) were merged as significant direct contributors to urinary 8-epi-PGF_2α_, and rs4607517, rs11558471, rs1387153, rs2166706, and rs10830963 tended to contribute to the association with urinary 8-epi-PGF_2α_ (Supplementary Table S6). In addition, all nine SNPs emerged as direct contributors to plasma MDA, except that rs2191349 only presented a tendency to contribute to the association (Supplementary Table S6).

## Discussion

In the present study, we examined nine susceptibility SNPs for IFG and newly diagnosed type 2 diabetes in a Korean population and constructed a wGRS to evaluate the influence of this genetic information on oxidative stress. The aggregation of common genetic variants influencing IFG and newly diagnosed type 2 diabetes plays a significant role in oxidative stress, despite having only small effects on glucose levels individually. wGRS was shown to be associated with the level of urinary 8-epi-PGF_2α_, which is the most reliable biomarker of lipid peroxidation and oxidative stress^11^. The urinary 8-epi-PGF_2α_ levels were significantly higher in the high-wGRS group than in the low- and middle-wGRS groups of subjects with IFG and newly diagnosed type 2 diabetes. Moreover, general population-level IV estimation (using wGRS as an instrument) strengthened the causal effect regarding the largely adverse influence of high levels of fasting serum glucose on markers of oxidative stress in the Korean population.

A highly significant correlation between blood glucose and urinary 8-epi-PGF_2α_ has been previously reported, suggesting that glycemic control is related to the determinants of lipid peroxidation^12^. Similarly, enhanced formation and release of 8-epi-PGF_2α_ by porcine vascular smooth muscle cells cultured under hyperglycemic conditions have been observed^13^. In the present study, the subjects with a high wGRS presented significantly higher serum glucose levels and HOMA-IR than those with a low wGRS in both the control and IFG and type 2 diabetes groups. Furthermore, the patients with IFG and newly diagnosed type 2 diabetes exhibited higher concentrations of urinary 8-epi-PGF_2α_ and plasma MDA, which is a lipid peroxidation biomarker^1^. Moreover, compared with the subjects in the low- and middle-wGRS groups, significantly higher concentrations of urinary 8-epi-PGF_2α_ and plasma MDA were observed in the IFG and newly diagnosed type 2 diabetes patients with a high wGRS. Thus, the higher fasting serum glucose and HOMA-IR observed in the subjects with high wGRS might be explained by elevated oxidative stress levels. Previous studies have demonstrated that oxidative stress plays the major role in IR pathogenesis^14,15^. There are several well-accepted mechanisms through which oxidative stress induces IR, including the activation of serine/threonine phosphorylation, disturbance of the cellular redistribution of insulin signaling components, decreased *GLUT4* gene transcription, and alteration of mitochondrial activity^16^. Indeed, further support is provided by studies showing that IR can be ameliorated by antioxidant treatment^17,18^.

In the current study, the IFG and type 2 diabetes patients showed steady increases in fasting serum glucose, insulin, HbA_1c_, and HOMA-IR across the wGRS groups. In addition, all these glucose-related markers were positively correlated with wGRS in the IFG and type 2 diabetes patients. In a previous study, Go *et al*.^19^ confirmed the cumulative incidence rates of type 2 diabetes according to the quartile-based GRS; moreover, fasting plasma glucose and HbA_1c_ steadily increased across the quartile-based GRS groups, which was consistent with the present study. In contrast to the IFG and type 2 diabetes patients, the NFG controls only showed a gradual increase in fasting serum glucose across the wGRS groups, which was additionally positively correlated with wGRS. Since wGRS was constructed using diabetes-related SNPs, fasting serum glucose and such glucose-related markers were positively correlated with wGRS. The high-wGRS group presented a higher potential risk probability for IFG and type 2 diabetes than the low-wGRS group because the high wGRS was constructed using the number of risk alleles with significantly high ORs. Therefore, the presence of a number of diabetes-related risk alleles could have more adverse effects on the risk of IFG and type 2 diabetes.

Polygenic risk scores have recently generated much interest for assessing the explanatory power of risk variants in the clinical management and prevention of type 2 diabetes^20^. However, currently available genetic information can only be used for predicting the development of type 2 diabetes, and the detailed relationship between GRS and the clinical features of type 2 diabetes is unclear. In the present study, we selected nine genetic variants associated with elevated fasting serum glucose levels in the Korean population and constructed a wGRS of IFG for type 2 diabetes development to evaluate the possibility that currently available genetic information can be translated into clinical practice. The constructed GRS (wGRS) showed a much stronger association with IFG and newly diagnosed type 2 diabetes than with any individual SNP. wGRS was also associated with the fasting serum glucose levels, HbA_1c_ levels, and HOMA-IR score in the IFG and newly diagnosed type 2 diabetes patients. Additionally, wGRS was strongly and significantly associated with the concentrations of urinary 8-epi-PGF_2α_ and plasma MDA in the IFG and newly diagnosed type 2 diabetes patients. Indeed, the causal relationship between fasting serum glucose and markers of oxidative stress was confirmed in the present study when wGRS was used as an IV. Thus, the combined effects of numerous common variants associated with slight effects on elevated fasting serum glucose are associated with the risk of oxidative stress.

In a recent study, both rs1260326 and rs780094 in *GCKR* were shown to be functional variants associated with type 2 diabetes and metabolic syndrome in a European population^21^. GCKR, which is a glucokinase regulatory protein produced in hepatocytes, binds glucokinase (GCK) and is involved in glucose metabolism^22^. Vaxillaire *et al*. showed that the *GCK* −30A allele is a true risk factor for the development of both IFG and type 2 diabetes, suggesting that this allele has a significant impact on β-cell function impairment^23^, which is consistent with our findings regarding rs1799884 in *GCK*. In the present study, only rs1260326 was detected on the Korean chip (K-CHIP) and was markedly associated with the risk of IFG and type 2 diabetes. *CDKN2A/B*, the cyclin-dependent kinase inhibitor 2 A/B gene, impacts diabetes risk across various ethnicities and geographical locations via β-cell mass and proliferation^24^. *MTNR1B*, a melatonin receptor gene, is a common variant associated with an increased risk of future type 2 diabetes and impaired early insulin secretion^25^. *DGKB*, the diacylglycerol kinase βgene, which regulates the intracellular concentration of the second messenger diacylglycerol, was also recently associated with fasting glucose and type 2 diabetes risk^26,27^. A previous functional study of *SLC30A8* suggested that reduced zinc transport increases type 2 diabetes risk^28,29^.

Oxidative stress plays a crucial role in the pathophysiology of type 2 diabetes and has been observed to be higher in type 2 diabetes patients than healthy controls^30^. The higher expression of the nine established SNPs observed under diabetic conditions in the present study might be governed by higher oxidative stress in diabetics. The first established gene/SNP in the current study, *GCKR*, which is associated with insulin-like growth factor-binding protein 1 (IGFBP1) levels, inhibits a glucokinase responsible for key functions such as glucose homeostasis and the conversion of glucose in pancreatic β-cells and liver hepatocytes^31^. The functional rs1260326 missense variant in *GCKR*, which is associated with decreased serum IGFBP1 and an increased risk of type 2 diabetes^31^. Oxidative stress, leading to reduced mammalian target of rapamycin (mTOR) signaling, may play a role in the development of IR^32^. Because IGFBP1 expression is strongly regulated by insulin, it serves as an excellent marker of IR in type 2 diabetes patients. Second, intracellular hyperglycemia promotes abnormal diacylglycerol (DAG) accumulation which induce the activation of protein kinase C (PKC). DAG is converted to phosphatidic acid (PA) by diacylglycerol kinase (DGK), and enhanced DGK activity leads to a reduction in excess DAG accumulation induced by high glucose. Atsumi *et al*.^33^ revealed that oxidative stress induced by high glucose can cause inhibition of DGK, possibly through redox modification of DGK. Based on a previous study, the T allele of rs2191349 near *DGKB* could lead to increased DGK activity, resulting in an excessive reduction of DAG levels, induced by hyperglycemic conditions. Third, possession of common risk alleles at the *SLC30A8* locus, encoding the β-cell granule zinc transporter, may affect Zn^2+^ concentrations and, thus, susceptibility to oxidative stress. Zinc is known for its antioxidative properties and has been extensively studied as a possible treatment option for diabetic patients^34,35^. Zinc supplementation was found to lower fasting glucose levels in carriers of the common type 2 diabetes risk allele at rs11558471^36^. Fourth, Kong *et al*.^24^ emphasized the roles of *CDKN2A/B* products and related proteins in the regulation of β-cell mass, proliferation, and insulin secretory function, in addition to roles in other metabolic tissues. Furthermore, a relationship between *CDKN2A/B* and oxidative stress has been reported, which is highly correlated with the hypermethylation of *CDKN2A/B*^37^. Last, melatonin receptors have been found throughout the body in many tissues and it has been shown that melatonin inhibits insulin secretion by pancreatic β-cells. Melatonin treatment improves BP, the lipid profile, and markers of oxidative stress in patients with metabolic syndrome^38^. The presence of copper ions could stimulate antioxidative stress mechanisms, including the synthesis of melatonin and its receptor. However, these mechanisms also likely include the activation of oxidative stress-related genes^39^. Taken together, the data indicate that the risk allele of *MTNR1B* is associated with a reduced antioxidative stress reaction due to a decrease in copper ions, which are involved in the synthesis of melatonin and its receptor. Therefore, melatonin cannot inhibit oxidation reactions catalyzed by reactive oxygen species scavengers, thus increasing fasting serum glucose as well as lipid peroxidation^40^.

The present study was the first to predict IFG and type 2 diabetes using a GRS based on the concentrations of 8-epi-PGF_2α_ and MDA, which are hallmarks of the intermediate phenotype of oxidative stress. In interpreting these findings, the typical limitations of cross-sectional observational studies should be considered, and these findings are indicative of the evaluated associations rather than prospective predictions. Additionally, we specifically focused on a representative group from the Korean population; thus, these results may not be generalizable to other ethnic groups. It could be more powerful to re-evaluate the nine identified SNPs in a replication cohort; therefore, a future investigation is warranted to define the causal variants within these loci to dissect their role in oxidative stress, which is a contributing factor for the development of IFG and type 2 diabetes. Despite these limitations, the present results show that the cumulative effects of common genetic variants associated with increased fasting serum glucose are also associated with oxidative stress, such as increases in the urinary 8-epi-PGF_2α_ and plasma MDA concentrations. The causal relationship between fasting serum glucose and markers of oxidative stress was confirmed through Mendelian randomization analysis. Therefore, this study indicates that a higher fasting serum glucose level could be an exact adverse effector of oxidative stress. As knowledge of genetic variation increases, preclinical genetic screening tools might enhance the prediction and prevention of clinical events.

## Methods

### Study population

In total, 2,113 study participants with NFG, IFG, and newly diagnosed type 2 diabetes between the ages of 20 and 86 years were recruited from the Health Service Center (HSC) during routine checkups at the National Health Insurance Corporation Ilsan Hospital in Goyang, Korea (January 2010–March 2015). Based on the data screened from the HSC, subjects presenting with IFG and type 2 diabetes were referred to the Department of Family Medicine or Internal Medicine, and their health and glucose profiles were then re-assessed. The diagnosis of diabetes or IFG was based on the fasting serum glucose levels (≥126 mg/dL or 100–125 mg/dL, respectively). The exclusion criteria included a current diagnosis and/or history of cardiovascular disease, liver disease, renal disease, pancreatitis, or cancer and the regular use of any medication. All the study participants provided written informed consent, and the Institutional Review Board of Yonsei University and the National Health Insurance Corporation of Ilsan Hospital approved the study protocol, which complied with the Declaration of Helsinki.

### SNP selection and genotyping

Based on a GWAS, 50 SNPs were established at type 2 diabetes loci showing the strongest associations with glycemic traits^10^. We selected 35 established SNPs associated with glycemic traits, particularly fasting glucose, including rs340874 near *PROX1*^26^; rs1260326 and rs780094 near *GCKR*^26,41^; rs11708067 and rs11717195 near *ADCY5*^26,41^; rs7651090 near *IGF2BP2*^42^; rs7708285 near *ZBED3*^42^; rs936822, rs7756992, and rs7747752 near *CDKAL1*^42–44^; rs17762454 near *SSAR1/RREB1*^42^; rs2191349 near *DGKB*^26^; rs1799884, rs6975024, rs4607517, and rs3757840 near *GCK*^26,42,43,45,46^; rs11558471 near *SLC30A8*^26,47^; rs7034200 near *GLIS3*^26^; rs10811661 near *CDKN2A/B*^42^; rs3829109 near *DNLZ*^42^; rs4506565, rs7903146, and rs12243326 near *TCF77 L2*^26,41,42,47^; rs11603334 near *ARAP1*^42,47,48^; rs1387153, rs10830963, and rs2166706 near *MTNR1B*^26,43,46,49–51^; rs116193319 and rs2293941 near *PDX1*^42,48^; rs17271305, rs4502156, and rs11071657 near *FAM148B/VPS13C/C2CD4A/B*^26,41,43,47^; and rs10423928, rs2302593, and rs11671664 near *GIPR*^41,42^.

Genotyping was performed using an Axiom^®^ 2.0 Reagent Kit (Affymetrix Axiom^®^ 2.0 Assay User Guide; Affymetrix, Santa Clara, CA, USA), and the genotype data were produced using the K-CHIP, which is available through the K-CHIP consortium. The K-CHIP was designed by the Center for Genome Science at the Korea National Institute of Health (4845–301, 3000–3031). The detailed procedure was described in a previous report^52^. Ten fasting glucose-related SNPs were not included in the K-CHIP; therefore, 25 SNPs were used for the subsequent analysis.

### GRS construction

Among the 25 SNPs, we constructed a wGRS using nine SNPs showing nominal significance (*p* < 0.05) and a consistent effect direction in the Korean population. Based on Bonferroni’s threshold (*p* < 0.002, 0.05 divided by 25), five SNPs remained after multiple testing. To maximize statistical power, all the SNPs with a nominal *p*-value <0.05 were included in the following risk score analysis. The estimate was analyzed by performing a logistic regression of the association between the number of risk alleles and the IFG and type 2 diabetes status. The wGRS was calculated by multiplying each estimated β-coefficient by the number of corresponding risk alleles (0, 1, or 2).

### Laboratory assessments

All anthropometry information has been previously described^53^. The participants’ weights and heights were measured to calculate their BMI (kilograms per square meter). The waist circumstance was measured to calculate the waist-to-hip ratio, and BP was assessed using an automatic BP monitor after a resting period of at least 20 min. After overnight fasting for at least 12 h, venous blood samples and urine samples were collected and stored at −80 °C and −20 °C, respectively. To assess the lipid profile, serum triglyceride, total cholesterol, and high-density lipoprotein (HDL) cholesterol levels were measured using commercial kits and an auto analyzer, and low-density lipoprotein (LDL) cholesterol levels were calculated using the Friedewald formula. The fasting serum glucose level and glucose-related markers, including insulin, HOMA-IR score, and HbA_1c_, were also measured. These laboratory assessments, including the lipid profile and glucose-related markers, have previously been described in detail^53^. Commercial assay kits were used to detect the urinary 8-epi-PGF_2α_ and plasma malondialdehyde (MDA) concentrations^54^.

### Statistical analysis

The statistical analyses, which included an independent t-test to compare two groups, determination of Pearson’s correlation coefficient to examine the relationships between wGRS and traits (variables), one-way ANOVA to compare three groups according to wGRS, a logistic regression to calculate the OR and 95% CI, a chi-square test to determine the frequency of the risk allele, and a general linear model UNIANOVA to adjust for confounding factors, were performed using SPSS v.24.0 (IBM/SPSS, Chicago, IL, USA). Skewed variables were log transformed, and all tests were considered statistically significant at a two-tailed *p*-value < 0.05. The causal effect of fasting serum glucose on markers of oxidative stress was analyzed under an IV regression with a two-stage least squares estimation method for all the subjects using wGRS as an instrument. In the first stage, the linear regression of fasting serum glucose on wGRS was calculated. In the second stage, the predicted values of fasting serum glucose were used as covariates in the linear regression analysis with markers of oxidative stress as the dependent variable. All the regressions were performed after adjustment for age, sex, and BMI.

## Electronic supplementary material

Supplementary information
