# Supplementary material for: Genetic risk score of common genetic variants for impaired fasting glucose and newly diagnosed type 2 diabetes influences oxidative stress
Source: Sci Rep. 2018 May 18;8:7828. doi: 10.1038/s41598-018-26106-z (PMC5959868; doi:10.1038/s41598-018-26106-z)
Supplement: Supplementary file 1 — Supplementary information [file 41598_2018_26106_MOESM1_ESM.pdf]

Genetic risk score of common genetic variants for impaired fasting glucose and newly diagnosed type 2 diabetes influences oxidative stress

Minjoo Kim,[1] Minkyung Kim,[1] Limin Huang,[2,3] Sun Ha Jee,[4] Jong Ho Lee[1-3]

[1]Research Center for Silver Science, Institute of Symbiotic Life-TECH, Yonsei University, Seoul, 03722, Korea

[2]Department of Food and Nutrition, Brain Korea 21 PLUS Project, College of Human Ecology, Yonsei University, Seoul, 03722, Korea

[3]National Leading Research Laboratory of Clinical Nutrigenetics/Nutrigenomics, Department of Food and Nutrition, College of Human Ecology, Yonsei University, Seoul, 037222, Korea

[4]Institute for Health Promotion, Graduate School of Public Health, Yonsei University, Seoul, 03722, Korea

Correspondence: Jong Ho Lee, PhD

Department of Food and Nutrition, College of Human Ecology, Yonsei University

50 Yonsei-ro, Seodaemun-gu, Seoul, 03722, Korea

Fax: +82-2-364-9605, Tel.: +82-2-2123-3122, E-mail: jhleeb@yonsei.ac.kr

Supplementary Table S1. Classification of three groups according to wGRS among NFG controls and IFG and type 2 diabetes subjects

group	Low		Middle		High	
wGRS value	< 1.5100		1.5100~2.0569		≥ 2..0570	
Subject	NFG	IFG & T2D	NFG	IFG & T2D	NFG	IFG & T2D
% (*n*)	37.2 (519)	25.5 (183)	33.9 (473)	31.6 (227)	28.9 (403)	42.9 (308)

All the participants were divided into three approximately equally sized strata according to the wGRS; low-GRS, middle-wGRS, and high-wGRS groups.

Supplementary Table S2. Characteristics of NFG group subdivided by wGRS into three groups

	**Low-wGRS (*n*=519)**	**Middle-wGRS (*n*=473)**	**High-wGRS (*n*=403)**	***p*-value**
Age (year)	48.4±0.50	48.2±0.54	47.2±0.56	0.235
BMI (kg/m^2)	23.9±0.13	23.8±0.14	23.5±0.15	0.119
Waist-to-hip ratio	0.88±0.00	0.88±0.00	0.88±0.00	0.759
Fasting serum glucose (mg/dL)§	85.9±0.33[b]	87.0±0.33[a,b]	87.8±0.36[a]	<0.001
Insulin (μIU/dL)§	9.1±0.19	9.04±0.19	9.12.±0.22	0.873
hbA1c (%)§	5.68±0.03	5.66±0.04	5.72±0.03	0.473
HOMA-IR§	1.94±0.04	1.95±0.04	1.99±0.05	0.962
8-epi-$PGF_{2\alpha}$ (pg/mg creatinine)§	1477.9±41.1	1499.9±41.6	1519.4±41.6	0.686
Malondialdehyde (nmol/mL)§	8.27±0.10	8.20±0.12	8.13±0.11	0.572

Mean ± SE. §tested by logarithmic transformation. *p* value derived from a one-way ANOVA among three groups. All letters indicating p<0.05 were derived from the Bonferroni *post hoc* test. Comparisons without a significant difference are indicated with the same letter, and significant differences are indicated with a different letter.

Supplementary Table S3. Characteristics of total study participants subdivided by wGRS into three groups

	**Low-wGRS (*n*=702)**	**Middle-wGRS (*n*=700)**	**High-wGRS (*n*=711)**	***p*-value**
Age (year)	49.6±0.44	49.7±0.43	49.6±0.42	0.962
BMI (kg/m^2)	24.2±0.11	24.1±0.12	24.1±0.12	0.930
Waist-to-hip ratio	0.89±0.00	0.89±0.00	0.89±0.00	0.977
Fasting serum glucose (mg/dL)§	93.9±0.78^{c}	96.7±0.80^{b}	102.1±0.88^{a}	<0.001
Insulin (μIU/dL)§	9.03±0.16	9.20±0.17	9.61±0.23	0.467
hbA1c (%)§	6.04±0.05^{b}	6.07±0.06^{b}	6.38±0.06^{a}	<0.001
HOMA-IR§	2.08±0.04^{b}	2.19±0.05^{b}	2.47±0.08^{a}	<0.001
8-epi-PGF$_{2\alpha}$ (pg/mg creatinine)§	1505.3±34.5^{b}	1534.0±35.8^{b}	1704.9±33.5^{a}	<0.001
Malondialdehyde (nmol/mL)§	8.90±0.13^{b}	8.88±0.14^{b}	10.3±0.22^{a}	<0.001

Mean ± SE. §tested by logarithmic transformation. *p* value derived from a one-way ANOVA among three groups. All letters indicating p<0.05 were derived from the Bonferroni *post hoc* test. Comparisons without a significant difference are indicated with the same letter, and significant differences are indicated with a different letter.

Supplementary Table S4. Ordinary least squares of fasting serum glucose to markers of oxidative stress

	Total (*n*=2,113)		*p*-value
	Standardized *β*	95% CI	
8-epi-$PGF_{2α}$ (pg/mg creatinine)[§]	0.097	0.041-0.112	<0.001
Malondialdehyde (nmol/mL)[§]	0.143	0.056-0.106	<0.001

95% CI, 95% confidence interval. [§]tested by logarithmic transformation. Beta coefficient was obtained from standard regressions with an ordinary least squares estimation method. All regressions were adjusted for age, sex, and BMI.

Supplementary Table S5. Instrumental variable estimates of fasting serum glucose to markers of oxidative stress based on interaction of the wGRS

	**Total (*n*=2,113)**		***p*-value**
	**Standardized *β***	**95% CI**	
8-epi-$PGF_{2\alpha}$ (pg/mg creatinine)[§]	0.295	1.026-1.920	<0.001
Malondialdehyde (nmol/mL)[§]	0.204	0.754-2.030	<0.001

95% CI, 95% confidence interval. [§]tested by logarithmic transformation. Beta coefficient was obtained from instrumental variable regressions with a two-stage least squares estimation method, using wGRS as an instrument for fasting glucose. All regressions were adjusted for age, sex, and BMI.

Supplementary Table S6. Association between specific 9 SNPs and markers of oxidative stress

	NFG (*n*=1,395) vs. IFG and type 2 diabetes (*n*=718)		*p*-value
	Standardized *β*	95% CI	
**Urinary 8-epi-PGF$_{2\alpha}$**			
rs1260326	0.033	-0.008-0.053	0.153
rs2191349	0.067	0.016-0.080	0.004
rs1799884	0.023	-0.019-0.060	0.313
rs4607517	0.042	-0.003-0.071	0.068
rs11558471	0.043	-0.001-0.060	0.061
rs10811661	0.061	0.011-0.074	0.008
rs1387153	0.043	-0.001-0.060	0.061
rs2166706	0.045	0.000-0.061	0.051
rs10830963	0.043	-0.002-0.059	0.063
**Plasma MDA**			
rs1260326	0.065	0.011-0.054	0.004
rs2191349	0.037	-0.004-0.042	0.098
rs1799884	0.077	0.001-0.021	0.001
rs4607517	0.073	0.018-0.070	0.001
rs11558471	0.081	0.019-0.062	<0.001
rs10811661	0.076	0.016-0.060	0.001
rs1387153	0.066	0.011-0.055	0.003
rs2166706	0.073	0.015-0.058	0.001
rs10830963	0.058	0.007-0.050	0.010

95% CI, 95% confidence interval. *p*-values were derived from a multiple regression analysis after adjusting for age, sex, and BMI.

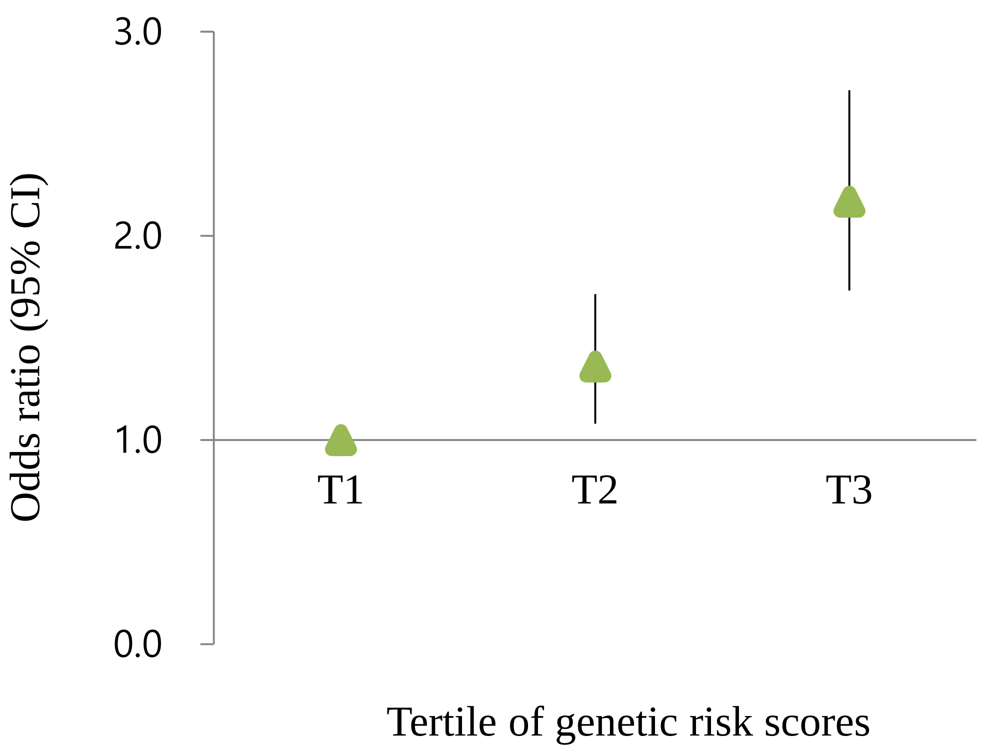

Supplementary Figure S1. Tertile-based odds ratio for IFG and type 2 diabetes risk allele

Odds ratio was calculated by logistic regression model. T1, low-wGRS; T2, middle-wGRS; T3, high-wGRS. T2: OR=1.361, 95% CI=1.080-1.715, T3: OR=2.168, 95% CI=1.732-2.713.
